# 1716. *In Vitro* Drug-Drug Interaction Evaluation of Epetraborole, a Novel Bacterial Leucyl-tRNA Synthetase Inhibitor

**DOI:** 10.1093/ofid/ofac492.1346

**Published:** 2022-12-15

**Authors:** Afshin Shafiee, Bradley K Wong, Sanjay Chanda

**Affiliations:** AN2 Therapeutics, Oakland, California; Wong DMPK Consulting, Redwood City, California; AN2 Therapeutics, Inc., Menlo Park, California

## Abstract

**Background:**

Epetraborole (EBO) is a boron-containing oral inhibitor of bacterial leucyl-tRNA synthetase, an essential enzyme in protein synthesis; EBO demonstrates potent activity against nontuberculous mycobacteria. EBO is being developed for the treatment of *Mycobacterium avium* complex lung disease patients and will be used in combination with other drugs. Therefore, EBO and its major circulating metabolite M3 were evaluated in a comprehensive drug-drug interactions (DDI) risk assessment.

**Methods:**

Stability of EBO was evaluated in human liver microsomes, hepatocytes and recombinant CYP enzymes. The inhibitory potential of EBO (0.03-100 μM) and M3 (1-1000 μM) on cytochrome P450 (CYP) activities was assessed using human hepatic microsomes. The CYP induction potential of EBO (25-200 μM), M3 was evaluated and compared to prototypical inducers in human hepatocytes (three donors) and mRNA. Fold increase in mRNA expression was utilized to investigate the CYP induction potential of EBO (0.3-100 µM) and M3 (1-250 µM). Stably transfected cell lines that expressed individual transporters were used to determine whether EBO or M3 were substrates or inhibitors for these proteins.

**Results:**

In vitro studies with microsomes, hepatocytes and recombinant cytochrome P450 (CYP) enzymes indicated that EBO was a poor substrate for major CYP enzymes; drug interactions with epetraborole as victim are considered unlikely. Neither EBO nor its major metabolite M3 was a potent reversible or time-dependent inhibitor of major CYP enzymes. Half maximal inhibitory concentration (IC_50_) values for CYP1A2, 2B6, 2C8, 2C9, 2C19, 2D6, 2E1 and 3A4 were >100 µM. EBO was not an inducer of CYP1A2 mRNA in human hepatocytes from three donors, while it was a weak inducer of CYP2B6 and CYP3A4. No induction of mRNA was observed in human hepatocytes at concentrations relevant to planned clinical doses. EBO is unlikely to be a substrate for P-gp, BCRP, OATP1B1, OATP1B3, OAT1, OAT3, MATE1 and MATE2K. EBO was an in vitro substrate for OCT2, a transporter involved in active renal secretion. At clinically relevant concentrations, neither EBO nor M3 inhibited major human efflux or uptake transporters.

**Conclusion:**

At clinically relevant concentrations of EBO and its major circulating metabolite M3, there is a low risk of victim or perpetrator DDI.

**Disclosures:**

**Afshin Shafiee, PhD**, AN2 Therapeutics: Stocks/Bonds **Bradley K. Wong, PhD**, AN2 Therapeutics: Advisor/Consultant **Sanjay Chanda, PhD**, AN2 Therapeutics: Stocks/Bonds.

